# Supplementary material for: Identifying impacts of contact tracing on HIV epidemiological inference from phylogenetic data
Source: Virus Evol. 2025 Sep 18;11(1):veaf068. doi: 10.1093/ve/veaf068 (PMC12481701; doi:10.1093/ve/veaf068)
Supplement: supplement_final_veaf068 [file supplement_final_veaf068.pdf]

Identifying Impacts of Contact Tracing on Epidemiological Inference from Phylogenetic Data

Supplementary material

Michael D. Kupperman[1,2], Ruian Ke [1], and Thomas Leitner[1,*]

August 1, 2025

1 Supplementary figures and tables

Table S1: Parameter values used to generate simulated data in SEEPS to approximate EU and Swedish outbreak samples subtree exterior/interior branch length ratio distributions. Transmission rate ratio is the proportion of the transmission rate in the first 3 months of infection, relative to the remaining average 21 months.

Parameter	EU simulation value	SE simulation value
Number of trials per parameterization	200	10,000
transmission rate ratio	20:1	1:1 (uniform)
Phase 1 length [Years]	2	1
Phase 1 R_0	3, 5	3, 5
Phase 1 maximum effective population size	7	100
Phase 2 length [Years]	2	5.5
Phase 2 R_0	2, 3, 4, 5, 6	2, 3, 4, 5
Phase 2 effective population size	600, 700, 800, 900, 1000	25, 30, 40, 50
Sampling time shift [Years]	18	0

[1] Theoretical Biology and Biophysics group, Los Alamos National Laboratory, Los Alamos, NM, USA
[2] Department of Applied Mathematics, University of Washington, Seattle, WA, USA

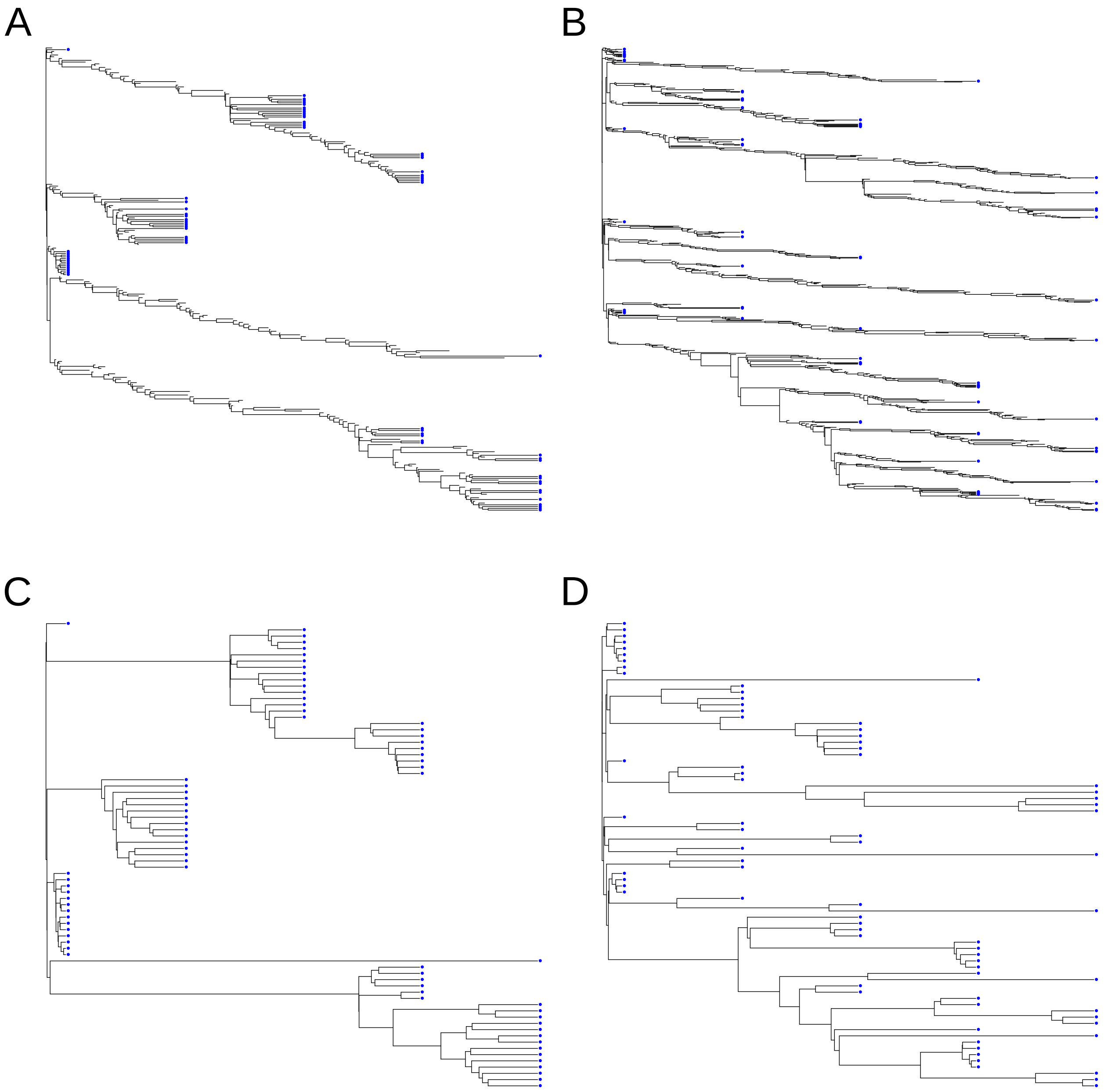

Fig. S1: Examples of how repeated sampling with different contact tracing levels can lead to visually distinct tree structures. Panel A (high contact tracing) and panel B (low contact tracing) show examples of two simulations provided by SEEPS. Note that SEEPS simulates within host diversity for all ancestors, resulting in explicit modeling of the transmission bottlenecks and the realistic population diversity. Panels C and D show only the "observable" history that could be inferred by reconstructing the ancestral relationships between the observed sequences. Sampled taxa are denoted by a blue circle.

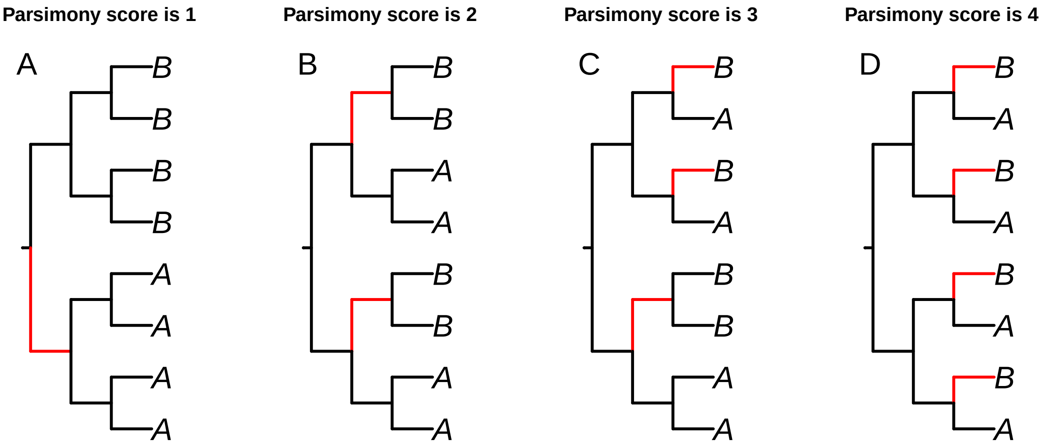

Fig. S2: Examples of how the 2-label parsimony is calculated. Each graph has 8 leaves: 4 labeled "A" and 4 "B". Since parsimony is a topological property, all distances have been standardized. The 2-label parsimony is the minimum number of "A" $\leftrightarrow$ "B" transitions necessary to explain the data. Also note that for the two-state problem, there is a symmetry to the problem, as a solution exists for either "A" $\rightarrow$ "B" or "B" $\rightarrow$ "A". For example, note that (A) places a "B" to "A" transition on the tree. An second, equivalent solution exists, placing a "A" to "B" transition on the opposite corresponding branch. Both solutions admit the same parsimony score (1). One possible solution to the optimization problem is shown above for each pattern of labels, where the branches containing a transition are marked in red. In (A), only a single "A" $\leftrightarrow$ "B" transition is needed (marked in red) to explain the data, so the 2-label parsimony score is 1. In (B), only two transitions (marked in red) are needed to explain the data, so the 2-label parsimony score is 2. Similarly, (C) requires 3 and (D) requires 4 transitions.

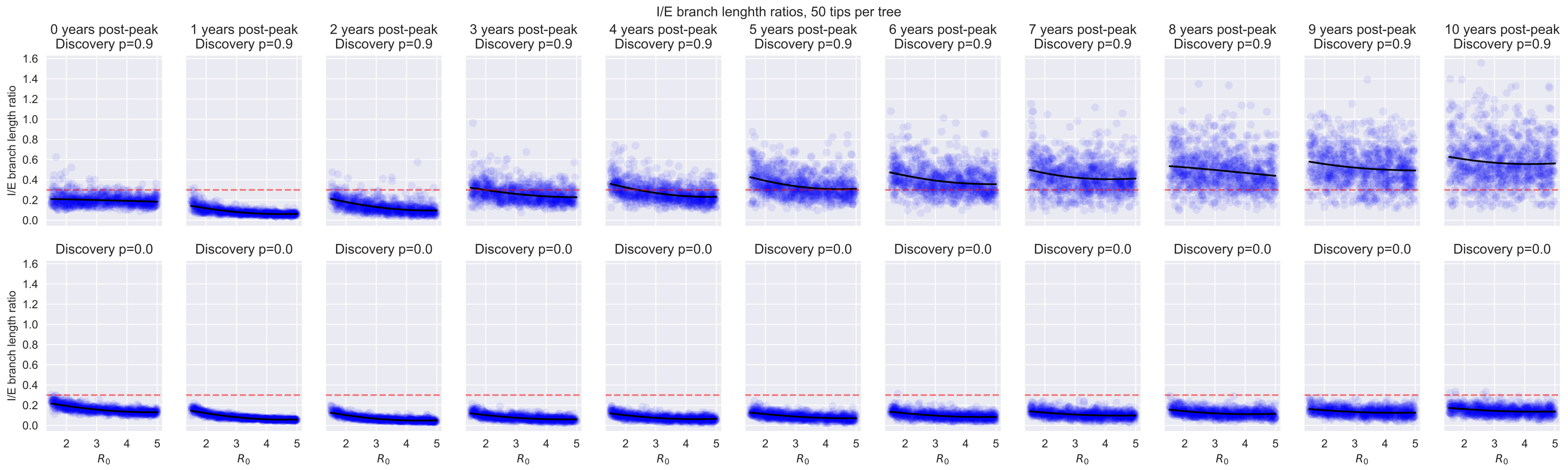

Fig. S3: Distribution of sampled I/E branch length ratios from independent samples taken between 0 and 10 years after the end of an exponentially growing outbreak, during a constant phase with R_0 randomized. The top row is generated with very high levels of contact tracing (discovery probability $p = 0.9$) and the bottom row is generated with very low levels of contact tracing (discovery probability $p = 0.1$). Iterative contact tracing with restarts is used in both cases. A simple regression model with a Gaussian kernel is used ($\alpha = 3 \times 10^{-5}$ with $\ell = 3$) to generate a mean trend line, which is plotted. A reference line at 0.3 is plotted. This ad-hoc threshold gives a good separation between the two resulting distributions at the 10-year mark.

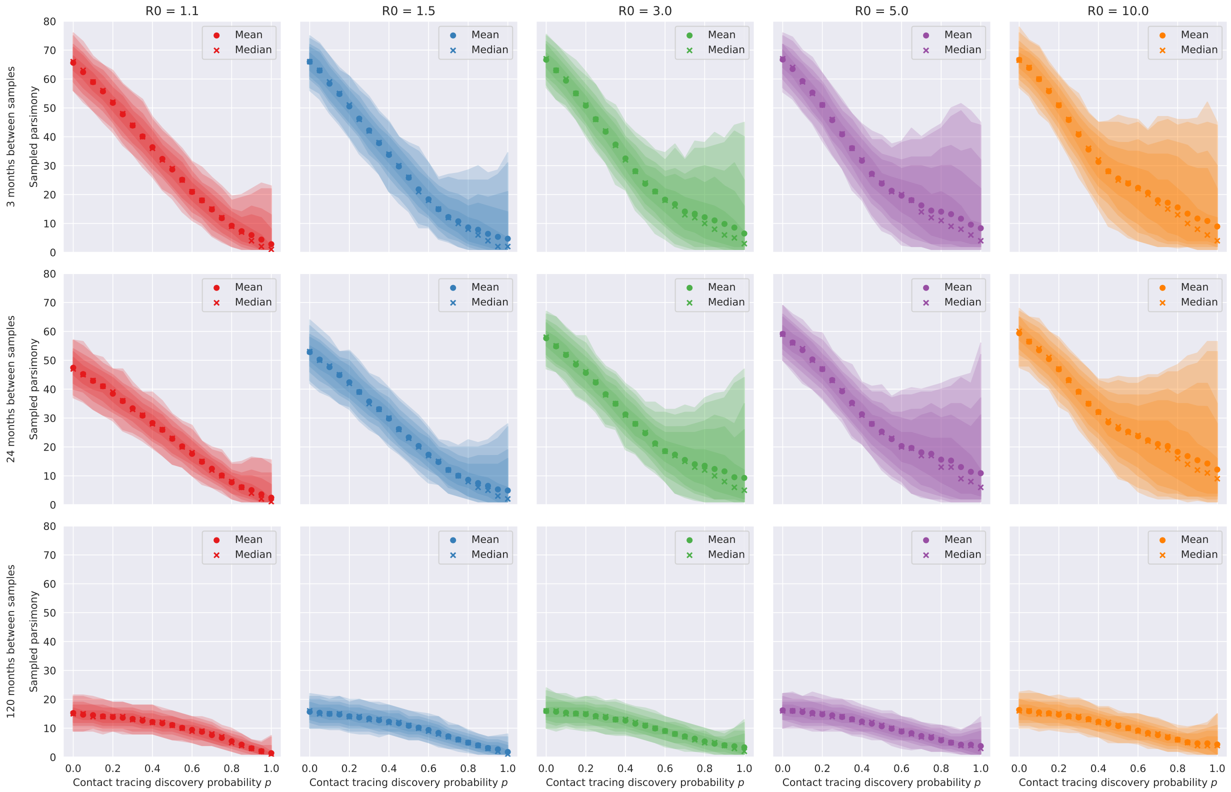

Fig. S4: More detailed representation of the parsimony distributions summarized in Fig. 4. Each panel shows the sampled parsimony distributions with contours drawn at the inner symmetric 99%, 98%, 90%, 80%, and 50% percentiles respectively. We can generally identify two regimes, partitioned into a *low* amounts of contact tracing and *high* amount of contact tracing. The amount of contact tracing in our model that separates the two regimes varies as R_0 changes. While there is an appreciable shift in the mean parsimony score as the contact tracing parameter increases, the two regimes are primarily identified through the change in spread.

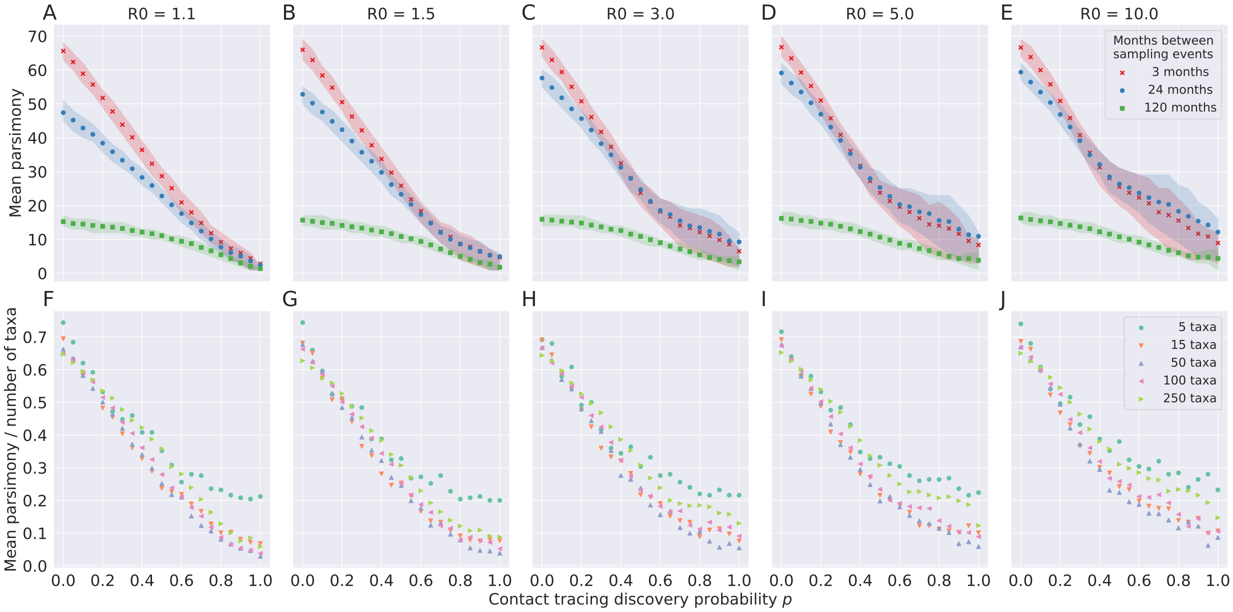

Fig. S5: Parsimony distributions are strongly related to contact tracing performance p and weakly with sample size. The strength of the correlation is primarily dependent on R_0 when contact tracing is good. In A-E, a sample of 100 taxa (pathogen sequences from 100 infected hosts) is obtained at the end of the exponential growth phase, and compared against another sample of 100 taken 3, 24, or 120 months later. The shaded region denotes the symmetric inner 50% of the data. In F-J, the experiment is repeated for the 3-month interval between samples, however the sample size is varied. The parsimony score is normalized against the number of taxa in the sampled tree (two times the reported sample size above). Sampled individuals are removed from the population.

2 Mathematics

2.1 Resampling branch lengths under a neutral model is minimally impactful

Consider a time-scaled tree T. We can generate distributions of new, closely related trees, $T' \sim F(T)$ by randomizing the tree T. In this appendix, we examine how rescaling the branches impacts the internal to external branch length ratio. We do not investigate here how altering the topology impacts the statistic.
A simple model for a mutation-scaled tree is to first simulate a time-scaled tree, then resample the branch lengths using a molecular clock, μ, which has units of substitutions per site per unit time.
These expected number of mutations are expanded into a distribution by assuming that the mutations accumulate under a Poisson distribution. Let the time distance d_i denote the length of the i'th branch length. Under this model, the expected number of mutations for a branch with length d_i is μd_i. This method is popular because it does not require any sequence specific knowledge (as would be necessary for TN93 or GTR based models) other than the length. SEEPS supports this method. For the time-scaled tree, the I/E branch length ratio is

$$\kappa(T) = \frac{\mathcal{I}(T)}{\mathcal{E}(T)} = \frac{\frac{1}{|\mathring{D}|}\sum\limits_{d_i \in \mathring{D}} d_i}{\frac{1}{|\bar{D}|}\sum\limits_{d_i \in \bar{D}} d_i} = \frac{|\bar{D}|}{|\mathring{D}|}\frac{\sum\limits_{d_i \in \mathring{D}} \mu d_i}{\sum\limits_{d_i \in \bar{D}} \mu d_i} \tag{1}$$

Where $\mathcal{I}(T)$ is the mean internal branch length of the tree T, $\mathcal{E}(T)$ is the mean external branch length of T, $\mathring{D}$ is the (indexed) set of internal branches, and $\bar{D}$ is the (indexed) set of external branches. For a fixed time-scaled tree, Poisson sampling the branch lengths results in a distribution,

$$\kappa(T') = \frac{|\bar{D}|}{|\mathring{D}|}\frac{\sum\limits_{d_i \in \mathring{D}} \text{Pois}(\mu d_i)}{\sum\limits_{d_i \in \bar{D}} \text{Pois}(\mu d_i)} = \frac{|\bar{D}|}{|\mathring{D}|}\frac{\text{Pois}\left(\mu \sum\limits_{d_i \in \mathring{D}} d_i\right)}{\text{Pois}\left(\mu \sum\limits_{d_i \in \bar{D}} d_i\right)} \tag{2}$$

We shall introduce some helpful shorthand. Let $X = \text{Pois}\left(\mu \sum\limits_{d_i \in \mathring{D}} d_i\right)$ and $Y = \text{Pois}\left(\mu \sum\limits_{d_i \in \bar{D}} d_i\right)$. We have $\mathbb{E}[X] = \text{Var}[X] = \mu \sum\limits_{d_i \in \mathring{D}} d_i$ and $\mathbb{E}[Y] = \text{Var}[Y] = \mu \sum\limits_{d_i \in \bar{D}} d_i$. By assumption, X and Y are independent, so $\text{Cov}(X, Y) = 0$. We can approximate the mean to second order as

$$\mathbb{E}\left[\frac{X}{Y}\right] \approx \frac{\mathbb{E}[X]}{\mathbb{E}[Y]} - \frac{\text{Cov}(X, Y)}{\mathbb{E}[Y]^2} + \frac{\mathbb{E}[X]\,\text{Var}(Y)}{\mathbb{E}[Y]^3} \tag{3}$$

So

$$\mathbb{E}[\kappa(T')] = \frac{|\bar{D}|}{|\mathring{D}|}\,\mathbb{E}\left[\frac{\text{Pois}\left(\mu \sum\limits_{d_i \in \mathring{D}} d_i\right)}{\text{Pois}\left(\mu \sum\limits_{d_i \in \bar{D}} d_i\right)}\right] \approx \frac{|\bar{D}|}{|\mathring{D}|}\left(\frac{\sum\limits_{d_i \in \mathring{D}} d_i}{\sum\limits_{d_i \in \bar{D}} d_i} + \frac{0}{\left(\mu \sum\limits_{d_i \in \bar{D}} d_i\right)^2} + \frac{\left(\mu \sum\limits_{d_i \in \bar{D}} d_i\right)\left(\mu \sum\limits_{d_i \in \mathring{D}} d_i\right)}{\left(\mu \sum\limits_{d_i \in \bar{D}} d_i\right)^3}\right) \tag{4}$$

$$= \frac{|\bar{D}|}{|\mathring{D}|}\frac{\sum\limits_{d_i \in \mathring{D}} d_i}{\sum\limits_{d_i \in \bar{D}} d_i} + \frac{|\bar{D}|}{|\mathring{D}|}\frac{0}{\left(\mu \sum\limits_{d_i \in \bar{D}}\right)^2} + \frac{|\bar{D}|}{|\mathring{D}|}\frac{\left(\mu \sum\limits_{d_i \in \bar{D}} d_i\right)\left(\mu \sum\limits_{d_i \in \mathring{D}} d_i\right)}{\left(\mu \sum\limits_{d_i \in \bar{D}} d_i\right)^3} \tag{5}$$

$$= \kappa(T) + \frac{|\bar{D}|}{|\mathring{D}|}\frac{\left(\mu \sum\limits_{d_i \in \mathring{D}} d_i\right)}{\left(\mu \sum\limits_{d_i \in \bar{D}} d_i\right)^2} = \kappa(T) + \frac{1}{\mu \sum\limits_{d_i \in \bar{D}} d_i}\left(\frac{|\bar{D}|}{|\mathring{D}|}\frac{\mu \sum\limits_{d_i \in \mathring{D}} d_i}{\mu \sum\limits_{d_i \in \bar{D}} d_i}\right) \tag{6}$$

$$= \kappa(T) + \frac{1}{\mu \sum\limits_{d_i \in \bar{D}} d_i}\kappa(T) = \kappa(T)\left(1 + \frac{1}{\mu \sum\limits_{d_i \in \bar{D}} d_i}\right) \tag{7}$$

$$= \kappa(T)\left(1 + \frac{|\bar{D}|}{|\bar{D}|\mu \sum\limits_{d_i \in \mathring{D}} d_i}\right) = \kappa(T)\left(1 + \frac{1}{|\bar{D}|}\frac{1}{\mu\mathcal{E}(T)}\right) = \kappa(T)\left(1 + \frac{1}{|\bar{D}|\mu}\frac{\kappa(T)}{\mathcal{I}(T)}\right) \tag{8}$$

giving an estimate of the increase in the I/E branch length ratio statistic when one re-samples edge lengths. We can further tidy this estimate if we assume that the tree is question is a binary tree.

Lemma 1. *For a tree T with N leaves, the number of interior branches, $|\mathring{D}|$, is $N-2$. The number of exterior branches, $|\bar{D}|$, is N.*

Using this lemma, we have

$$\mathbb{E}[\kappa_\mu(T')] \approx \kappa(T)\left(1 + \frac{1}{|\bar{D}|}\frac{1}{\mu\mathcal{E}(T)}\right) = \kappa(T)\left(1 + \frac{1}{N}\frac{1}{\mu\mathcal{E}(T)}\right) \tag{9}$$

Biologically, this tells us that that the average value of the distribution will always be greater than the original value of the statistic, when we resample the edge lengths of a tree. This effect is smallest when the the mutation rate is large, the number of interior branches is large, and when the mean exterior branch length is large.

We can also ask about the variance of the resulting distribution. A first order estimate of the variance follows as

$$\text{Var}(X/Y) \approx \frac{\mathbb{E}[X]^2}{\mathbb{E}[Y]^2}\left(\frac{\text{Var}(X)}{\mathbb{E}[X]^2} + \frac{\text{Var}(Y)}{\mathbb{E}[Y]^2} - 2\frac{\text{Cov}(X,Y)}{\mathbb{E}[X]\,\mathbb{E}[Y]}\right) \tag{10}$$

$$\begin{aligned}
\mathrm{Var}\left(\kappa_\mu(T')\right) &= \left(\frac{|\bar{D}|}{|\mathring{D}|}\right)^2 \mathrm{Var}\left(\frac{X}{Y}\right) && (11)\\
&\approx \left(\frac{|\bar{D}|}{|\mathring{D}|}\right)^2 \kappa(T)^2 \left(\frac{\mathbb{E}[X]}{\mathbb{E}[X]^2} + \frac{\mathbb{E}[Y]}{\mathbb{E}[Y]^2} - 2\frac{0}{\mathbb{E}[X]\,\mathbb{E}[Y]}\right) && (12)\\
&= \left(\frac{|\bar{D}|}{|\mathring{D}|}\right)^2 \kappa(T)^2 \left(\frac{1}{\mathbb{E}[X]} + \frac{1}{\mathbb{E}[Y]}\right) && (13)\\
&= \left(\frac{|\bar{D}|}{|\mathring{D}|}\right)^2 \kappa(T)^2 \left(\frac{1}{\left(\mu \sum\limits_{d_i \in \mathring{D}} d_i\right)} + \frac{1}{\left(\mu \sum\limits_{d_i \in \bar{D}} d_i\right)}\right) && (14)\\
&= \left(\frac{|\bar{D}|}{|\mathring{D}|}\right)^2 \kappa(T)^2 \left(\frac{1}{|\mathring{D}|}\frac{|\mathring{D}|}{\left(\mu \sum\limits_{d_i \in \mathring{D}} d_i\right)} + \frac{1}{|\bar{D}|}\frac{|\bar{D}|}{\left(\mu \sum\limits_{d_i \in \bar{D}} d_i\right)}\right) && (15)\\
&= \left(\frac{|\bar{D}|}{|\mathring{D}|}\right)^2 \kappa(T)^2 \left(\frac{1}{\mu|\mathring{D}|}\frac{1}{\mathcal{I}(T)} + \frac{1}{\mu|\bar{D}|}\frac{1}{\mathcal{E}(T)}\right) && (16)\\
&= \frac{\kappa(T)^2}{\mu}\frac{|\bar{D}|}{|\mathring{D}|^2}\left(\frac{|\bar{D}|}{|\mathring{D}|}\frac{1}{\mathcal{I}(T)} + \frac{1}{\mathcal{E}(T)}\right) && (17)
\end{aligned}$$

For a large full binary tree, $|\bar{D}| \approx |\mathring{D}|$. So the variance is a weighted scaling of the harmonic mean of the mean internal and external branch lengths. We can refine this estimate using lemma 1, to obtain more concrete estimates. These are summarized in the following theorem.

Theorem 2. *If T is a binary tree with N leaves, let $T' \sim F(T)$ be the distribution of possible trees generated by resampling edge distances at rate μ. Then the expected value of the mean interior-to-exterior branch length ratio is (to second order)*

$$\mathbb{E}[\kappa(T')] = \kappa(T)\left(1 + \frac{1}{N\mu}\frac{1}{\mathcal{E}(T)}\right) \tag{18}$$

and the variance is (to first order)

$$\mathrm{Var}\left(\kappa_\mu(P(T))\right) = \frac{\kappa(T)^2 N}{\mu(N-2)^2}\left(\left(\frac{N}{N-2}\right)\frac{1}{\mathcal{I}(T)} + \frac{1}{\mathcal{E}(T)}\right) \tag{19}$$

Let us provide a concrete example. Consider a time-scaled phylogenetic tree T of 50 tips/sequences ($N = 50$), where the average exterior branch length is 1 year and the average interior branch length is 2 years. For a sequence of 390 nucleotides (corresponding to the "V3-clinical" region in SEEPS) with a clock rate of 0.0067 substitutions per site per year. This gives $\mu = 0.0067 \cdot 390 = 2.613$ substitutions per year. If we Poisson resampled the branch lengths for this tree, we would expect the resampled distribution to have mean and variance

$$\mathbb{E}[\kappa(T')] = \frac{2}{1}\left(1 + \frac{1}{50 \cdot 2.613}\frac{1}{1}\right) = 2(1.00031942248) = 2.00063884 \approx \kappa(T)$$

$$\mathrm{Var}[\kappa(T')] = \frac{2^2(50)}{(2.613)(48)^2}\left(\left(\frac{50}{48}\right)\frac{1}{2} + \frac{1}{1}\right) = 0.05052307$$

While these are only estimates of the mean and variance to second and first order respectively, we see that for reasonably large trees under reasonable assumptions, the resulting distribution for $\kappa(T')$ is centered very close to $\kappa(T)$, with $1\sigma = \sqrt{\mathrm{Var}(\kappa(T'))} \approx 0.2247$, around 11% of $\kappa(T)$.

2.2 Adding a sampling delay gives predictable impacts on the model

Another common variation to consider is the addition of some sampling delay after the commencement of a contract tracing investigation. In this work, contact tracing is done cross-sectionally, at a fixed time point. However, even if the initial investigation can be done quickly, there may be a delay in collecting an associated sequence. Suppose that this waiting time, S, is the same distribution for each individual. That is, each sample has waiting time $s_i \sim S_i = S$. The distribution S is a waiting time distribution, it is conditioned on the individual being detected and being entered into the database. As such, we require that $\mathbb{P}(S < \infty) = 1$, and find the technical requirement $\mathbb{E}[S] < \infty$ sufficient.

Since the sampling time lag occurs after the patient is identified through contact tracing, this must extend the external branch associated with each individual, regardless of it's length. Thus, the apparent external branch for the i'th individual is $\tilde{d_i} = d_i + s_i$, where $s_i \sim S_i$ for $d_i \in \bar{D}$. The internal branches are unchanged. We denote the modified tree by $\tilde{T} \sim F(T)$. If this occurs for every sampled individual, the new I/E branch length is given by

$$\kappa(\tilde{T}) = \frac{\mathcal{I}(\tilde{T})}{\mathcal{E}(\tilde{T})} = \frac{\mathcal{I}(T)}{\frac{1}{|\bar{D}|}\sum\limits_{d_i \in \bar{D}} \tilde{d_i}} = \frac{\mathcal{I}(T)}{\frac{1}{|\bar{D}|}\sum\limits_{d_i \in \bar{D}} \tilde{d_i} + \frac{1}{|\bar{D}|}\sum\limits_{i=1}^{|\bar{D}|} s_i} = \frac{\mathcal{I}(T)}{\mathcal{E}(T) + \frac{\sum_{i=1}^{|\bar{D}|} s_i}{|\bar{D}|}} \tag{20}$$

We define the sample mean $\hat{s} \equiv \frac{\sum_{i=1}^{|\bar{D}|} s_i}{|\bar{D}|}$, and note that the central limit theorem admits an approximation for $\hat{s}$ as normally distributed (in the limit as the number of taxa/tips in the tree grows), with mean $\mathbb{E}[S]$ and variance $\mathrm{Var}(S)/\sqrt{|\bar{D}|}$. In practice, we find $|\bar{D}| = 50$ taxa is sufficient to observe limiting behavior. This argument omits one key fact - waiting times are always non-negative, so with probability 1, $\hat{s} \geq 0$.

This gives the limiting distribution for $\kappa(\tilde{T})$ as (approximately)

$$\kappa(\tilde{T}) = \frac{\mathcal{I}(T)}{\mathcal{E}(T) + \mathcal{N}(\mathbb{E}[S], \mathrm{Var}(S)/\sqrt{|\bar{D}|})} \tag{21}$$

We can estimate the behavior of this distribution under the assumption that $|\bar{D}| \to \infty$ using a first order taylor series expansion. Define $g(x;T) = \frac{\mathcal{I}(T)}{\mathcal{E}(T)+x}$, so $g'(x;T) = -\frac{\mathcal{I}(T)}{(\mathcal{E}(T)+x)^2}$. Then

$$\mathbb{E}[\kappa(\tilde{T})] = \mathbb{E}[g(\mathcal{N}(\mathbb{E}[S], \mathrm{Var}(S)/\sqrt{|\bar{D}|}))] \approx g(\mathbb{E}[S];T) = \frac{\mathcal{I}(T)}{\mathcal{E}(T) + \mathbb{E}[S]} \tag{22}$$

along with the first order estimate of the variance,

$$\mathrm{Var}\,\kappa(\tilde{T}) = \mathrm{Var}\,g(\mathcal{N}(\mathbb{E}[S], \mathrm{Var}(S)/\sqrt{|\bar{D}|})) \approx g'(\mathbb{E}[S];T)^2 \mathcal{N}(\mathbb{E}[S], \mathrm{Var}(S)/\sqrt{|\bar{D}|})) = \frac{\mathcal{I}(T)^2}{(\mathcal{E}(T) + \mathbb{E}[S])^4} \frac{\mathrm{Var}[S]}{\sqrt{|\bar{D}|}} \tag{23}$$

Thus, we can reasonably understand how the expended value of the I/E branch length ratio will be impacted for large trees, when a sampling delay is added. To summarize, for a sufficiently large phylogenetic tree, the expected branch length ratio is described by $\frac{\mathcal{I}(T)}{\mathcal{E}(T)+\mathbb{E}[S]}$. The choice of the lag distribution S is essential. A simple model for S is to choose S to be an exponential distribution, with a prescribed mean. Let us give an example.

With a 1 month average lag between detection and sequencing (which we presume to be a rather high estimate), we have $\mathbb{E}[S] = 1$ with $\mathrm{Var}(S) = 1$. Further suppose there are 50 taxa. This gives an estimate of $\mathbb{E}[S] = 1$ and $\mathrm{Var}(S)/\sqrt{50} \approx 0.1414$.

If we consider the example used to calculate the branch length ratios above where the average exterior branch is 1 year (12 months), and the average interior branch is 2 years (24 months). Adding this lag reduces the observed I/E branch length ratio, however only from 2 to $\frac{24}{12+1} \approx 1.846$, a reduction of approximately 8%.
